# Veneer type determines shear bond strength to CAD/CAM multilayer glass-fiber–reinforced polymer frameworks after thermomechanical aging

**DOI:** 10.1186/s12903-025-07247-w

**Published:** 2025-12-07

**Authors:** Habibe Öztürk Ulusoy, Hasan Murat Aydoğdu, İlgi Tosun

**Affiliations:** 1 Department of Prosthodontics, Çanakkale Onsekizmart University, Çanakkale, Türkiye; 2 Department of Prosthodontics, Çanakkale Onsekizmart University, Dişhekimliği Fakültesi, Kepez, Sahilyolu Cd No:5, Çanakkale, TR 17110 Türkiye

**Keywords:** Fiber-reinforced polymer, CAD/CAM, Shear bond strength, Composite resin hybrid ceramic, Thermomechanical aging

## Abstract

**Background:**

This study tested the null hypothesis (H0) that the type of veneering material would have no effect on the shear bond strength (SBS) to multilayer glass-fiber-reinforced polymer (MLG-FRP) frameworks.

**Methods:**

Eighty-four specimens were prepared from Trinia and Zantex CAD/CAM discs (10 mm diameter, 1 mm thickness) and divided into six subgroups (*n* = 14). Each subgroup was veneered with composite resin (Ceramage, CE), milled hybrid ceramic (CeraSmart, CS), or 3D-printed hybrid ceramic (VarseoSmile Crown Plus, VS). All specimens underwent thermomechanical aging (240,000 chewing cycles; 5000 thermal cycles at 5–55 °C). SBS was measured using a universal testing machine, and failure modes were classified under a stereomicroscope (20×). Premature test failures (PTFs) were coded as 0 MPa in the ITT analysis, with an additional PP analysis excluding PTFs, and data were analyzed by two-way ANOVA with Tukey’s post-hoc test (α = 0.05) including Shapiro–Wilk, Levene, partial η², and Hedges g (95% CI).

**Results:**

The null hypothesis that veneering material does not affect bond strength was rejected. Veneering material significantly affected shear bond strength (SBS) (ITT: F = 5.66, *p* = 0.005; PP: F = 4.30, *p* = 0.018), while MLG-FRP type and interaction were not significant (*p* > 0.05). In intention-to-treat analysis (PTFs = 0 MPa), composite resin (CE: 12.87 ± 2.66 MPa; 95% CI: 11.88–13.86) outperformed milled (CS: 9.19 ± 6.16 MPa) and 3D-printed hybrids (VS: 9.02 ± 4.74 MPa). Per-protocol analysis confirmed this trend (CE: 12.87 ± 2.66 MPa > CS: 12.87 ± 2.04 ≈ VS: 10.99 ± 2.25). Effect sizes were large (Hedges g up to 1.2). Kaplan–Meier analysis showed 100% survival in CE, significantly higher than CS (*p* = 0.002) and VS (*p* = 0.02), with no difference between CS and VS. Failures were mainly adhesive (> 90%).

**Conclusions:**

The choice of veneering material is a critical determinant of bond strength to MLG-FRP frameworks. Composite resin provided significantly stronger and more durable adhesion than hybrid ceramics under simulated aging, supporting its use for long-term clinical reliability.

**Supplementary Information:**

The online version contains supplementary material available at 10.1186/s12903-025-07247-w.

## Introduction

 The field of restorative dentistry has witnessed significant advancements with the advent of computer-aided design/computer-aided manufacturing (CAD/CAM) technologies. These innovations have enabled the production of high-precision, metal-free dental restorations, which are increasingly preferred due to their biocompatibility, aesthetics, and mechanical properties. Among the materials used in CAD/CAM systems, dental ceramics and zirconia have gained widespread popularity as alternatives to traditional metal alloys. However, despite these advantages, these restorations can still present failures such as veneer chipping and delamination, which complicate clinical longevity and repair procedures [1].

In recent years, fiber-reinforced polymers (FRPs) have emerged as promising non-metallic materials for fixed and removable prosthetic frameworks. FRPs offer high biocompatibility, a low elastic modulus, and excellent fracture resistance, making them suitable for both cemented and screw-retained restorations [2–4]. Multilayer glass-fiber-reinforced polymers, such as Trinia and Zantex, comprise a polymer matrix reinforced with a three-dimensional glass fiber network. They have garnered attention for their favorable mechanical performance and compatibility with CAD/CAM milling technologies. However, due to their limited optical properties, MLG-FRPs are not suitable for monolithic restorations. To overcome this limitation, a veneering material is applied to mask the fibers, enhance esthetics, prevent soft tissue irritation, and reduce plaque accumulation associated with surface roughness [5, 6]. Traditionally, composite resins have been used as veneering materials for FRP frameworks; however, studies have reported relatively high failure rates, including fractures and delamination like veneered zirconia, with incidences of such complications reaching up to 41% [7].

Resin-based hybrid ceramics, a new class of hybrid materials, combine the structural durability and color stability of ceramics with the low abrasiveness and superior flexural properties of polymers [8]. These materials are increasingly utilized in CAD/CAM systems for fabricating durable single- and multi-unit restorations [9]. Cerasmart, a nanoparticle-filled hybrid ceramic resin, exhibits excellent flexural strength and a Young’s modulus comparable to dentin, reducing the risk of fracture and chipping [10]. Similarly, VarseoSmile Crown Plus, a recently introduced 3D-printable hybrid composite resin, provides remarkable dimensional stability, flexural strength, and esthetics [11]. Despite growing clinical use, there is a notable gap in research on their application as veneering materials for FRP frameworks, as existing studies have primarily focused on conventional composite resins [12, 13].

Although manufacturers claim that composite resins used in FRC-fixed partial dentures are less prone to fracture and chipping, recent clinical data indicate otherwise. A retrospective analysis revealed a notably high incidence (41%) of technical complications, including failures, fractures, and delamination, primarily attributed to the composite resin component [7]. These adverse outcomes may be linked to manual fabrication techniques, where void formation during hand-layering increases defect density and compromises the structural integrity of the material [14]. In contrast, the advent of CAD/CAM technology has markedly improved the fabrication process. Restorative materials produced industrially under controlled temperature and pressure conditions exhibit significantly fewer defects and demonstrate enhanced resistance to fracture and chipping, thereby expanding their clinical applicability [3, 15].

Therefore, the present study was designed to comprehensively assess the bonding efficacy of various veneering materials—namely milled hybrid ceramics (GC Cerasmart), 3D-printed hybrid ceramics (VarseoSmile Crown Plus), and conventional composite resins—when applied to multilayer glass-fiber-reinforced polymers subjected to simulated clinical aging protocols. The null hypothesis posited that there would be no significant difference in the bond strength of composite resin, milled, and 3D-printed hybrid ceramic materials to MLG-FRP frameworks. By addressing this notable gap, the study aims to inform evidence-based material selection for MLG-FRP restorations, thereby enhancing the predictability and longevity of clinical outcomes.

## Materials and methods

### Sample size calculation

The sample size was determined using G*Power version 3.1.9.6 (Universitat Kiel, Kiel, Germany) based on the maximum effect size observed for shear bond strength between the control and Al₂O₃ surface treatment groups in the study by Kürkçüoğlu et al. [13]. Although that study focused on surface treatments rather than veneering material differences, the similar test method and interface justified its use as a conservative basis for estimating the required sample size. To accommodate experimental variability and potential testing failures, a sample size of 14 specimens per test group was established.

### Fabrication of specimens

**Fig. 1:**
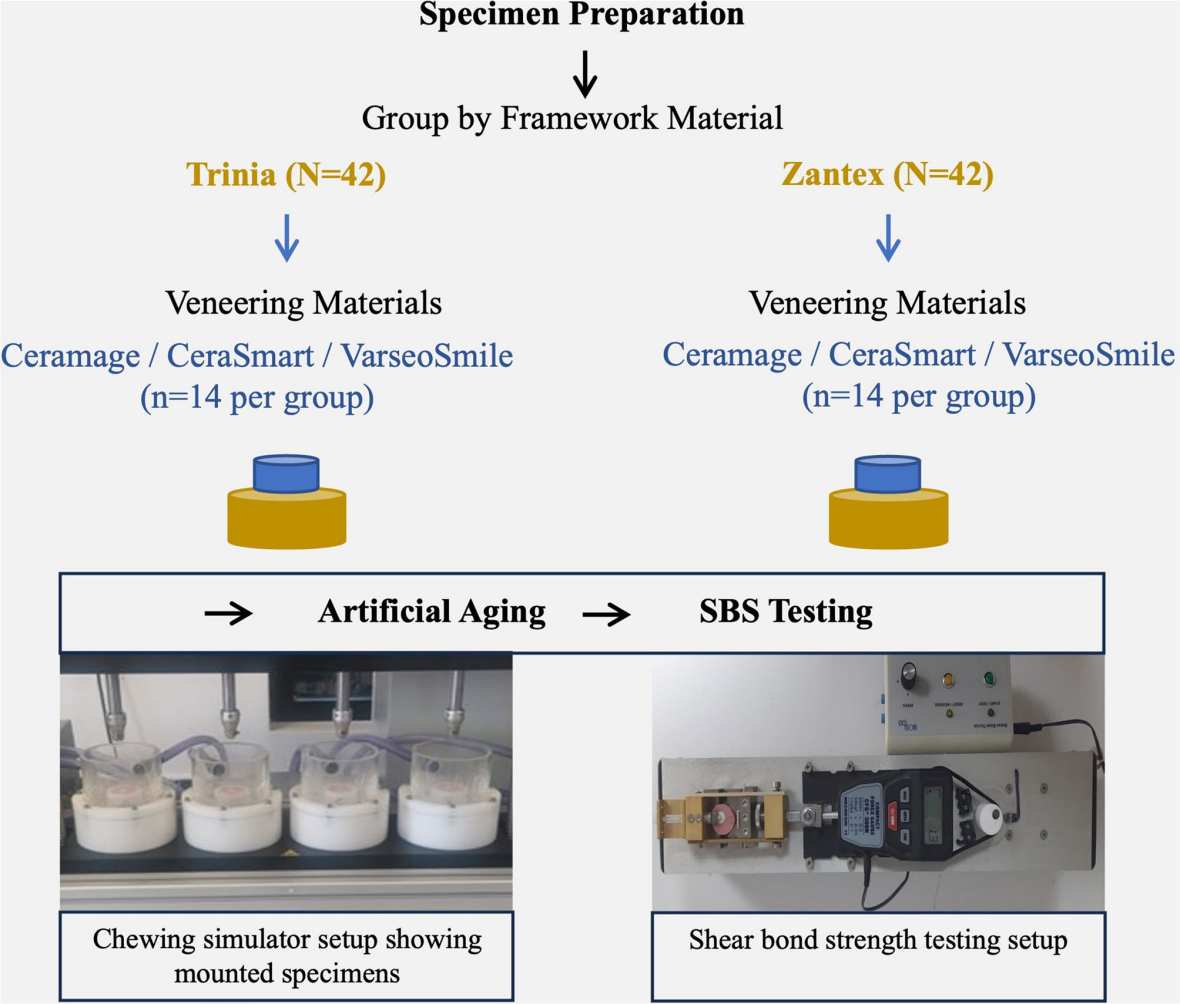
Study workflow diagram: experimental design, the specimen preparation, artificial aging, and SBS testing

Figure 1. illustrates the comprehensive experimental workflow, while Table 1 provides detailed specifications of all materials used in this investigation. The dimensions of the veneering material (3 mm diameter and 4 mm height) were selected in accordance with the ISO 29022:2013 standard for notched-edge shear bond strength testing, to provide standardized bonded areas and homogeneous stress distribution at the interface [16].

**Table 1:** Material properties and compositions of the materials utilized during the investigation

Material Type	Name		Composition	Manufacturer
MLG-FRPs		*Trinia (T)*	60% glass-fiber, 40% epoxy resin	Shofu, Kyoto, Japan
		*Zantex (Z)*	45% glass-fiber, 28% epoxy resin*	Biofunctional Materials, Boca Raton, USA
Veneering Materials		*Ceramage (CE)*	Light-curing micro-hybrid composite	Shofu, Kyoto, Japan
		*CeraSmart (CS)*	71% silica (20 nm), barium glass (300 nm), Bis-MEPP, UDMA, DMA	GC Europe, Leuven, Belgium
		*VarseoSmile Crown Plus (VS)*	Ceramic-filled (30–50 wt% inorganic fillers; particle size 0.7 μm) silanized dental glass, methyl benzoyl formate	Bego, Bremen, Germany
Bonding Agent		*Ceraresin*	Bond I: silane coupling agent, ethanol; Bond II: 4-MET, UDMA, polymerization initiator, acetone	Shofu, Kyoto, Japan
Luting Cement		*Panavia V5*	10-Methacryloyloxydecyl dihydrogen phosphate (MDP), 2-hydroxyethyl methacrylate (HEMA), hydrophilic aliphatic dimethacrylate, accelerators, water	Kuraray Noritake, Tokyo, Japan

*Bis-MEPP* 2,2-bis 4-methacryloxypolyethoxyphenyl propane, *UDMA* urethane-dimethacrylate, *DMA* dimethacrylate, *4-MET* 4-methacryloxyethyl trimellitic anhydride

*Based on the characterization findings of Bergamo [2], the manufacturer did not provide any data regarding the composition of the material

#### CAD-CAM glass fiber reinforced high-performance polymer

CAM software (SolidWorks Corporation, Dassault Systèmes S.A, Waltham, USA) was utilized to design discs with dimensions of 10 mm in diameter and 1 mm in thickness. Forty-two Trinia (98 mm diameter) and forty-two Zantex (98 mm diameter) cores were produced using a CAD/CAM milling unit (Ceramill Motion 2, Amann Girrbach, Koblack, Austria). These were divided into three groups according to veneering material (*n* = 14).

#### CAD/CAM millable nanohybrid ceramic resin — CeraSmart (CS)

For the subtractively manufactured group, a cylindrical STL file (3 × 4 mm) was prepared and transferred to the nesting software (**SolidWorks Corporation**,** Dassault Systèmes S.A**,** Waltham**,** USA**). The same milling unit was employed to mill the CeraSmart specimens to the desired dimensions.

#### 3D printable nanohybrid composite resin — VarseoSmile Crown Plus (VS)

The identical STL design used for the CS group was imported into the BegoCAM Creator Print software (Bego GmbH, Bremen, Germany) and positioned on the build platform. After automatic generation of supports, printing was performed with a DLP printer (Varseo XS, Bego GmbH, Bremen, Germany). Post-processing included ultrasonic cleaning in reusable 95% isopropyl alcohol. The post-processing involved removal of support structures and subsequent 50 μm glass-bead cleaning. Importantly, this cleaning was limited to the external support regions and did not contact the bonding surface, thereby avoiding potential confounding effects on adhesion. Final polymerization was performed using a xenon flash unit (Bego Otoflash, Bego GmbH, Bremen, Germany).

### Luting of specimens

Prior to bonding, all specimens were air-abraded with 110 μm Al₂O₃ particles at ~ 2 bar pressure for 15 s, applied at a distance of 10 mm and perpendicular to the bonding surface [13]. A tape with a 3 mm diameter hole was used to standardize the cementation area. Bonding was carried out according to the veneering material applied to each group.

#### Veneer composite resin — Ceramage (CE)

Following surface preparation, a primer (Ceraresin Bond 1 and 2) was applied with a micro brush and left for 10 s. Polymerization was performed for 20 s using a calibrated LED curing unit (Woodpecker Led-C, Guilin, Guangxi, China). A silicone index (Memosil Transparent Bite Registration Material, Kulzer, Hanau, Germany) with an internal 3 × 4 mm cavity ensured standardized thickness, and Ceramage composite was applied manually as per the manufacturer’s recommendations.

#### Resin-matrix ceramic systems (CS and VS groups)

For the CS and VS groups, Panavia V5 resin cement was applied under constant pressure after appropriate surface treatment. Each cement layer was light-cured for 20 s using the same LED unit at a maximum intensity of 1200 mW/cm². The bonded specimens were then stored in distilled water at 37 °C for 24 h.

### Aging and fatigue testing

Artificial aging was performed using a chewing simulator (SD Mechatronik, Feldkirchen-Westerham, Germany) with a total of 240,000 cycles at a load of 50 N and a frequency of ~ 1.2 Hz. Each cycle involved vertical loading with a dwell time of 1.5 s in distilled water at 37 °C. Steatite balls (6 mm diameter) were used as antagonists. The loading path was vertical without lateral sliding to simulate masticatory forces [17]. Simultaneously, thermocycling was performed between 5 °C and 55 °C in distilled water baths, with each cycle lasting 30 s during a total of 5000 cycles (Fig. 1). These parameters simulated approximately one year of clinical service. Specimens were monitored three times daily throughout testing to record failures for survival analysis.

Following artificial aging, shear bond strength (SBS) was tested according to ISO 29022:2013 (notched-edge method) using a universal testing machine [16]. A 0.7 mm diameter stainless steel wire was applied at the adhesive interface at a crosshead speed of 0.50 mm/min until failure (Fig. 1). The bonded area (A) was calculated as πr², and SBS was obtained by dividing the load at failure (N) by this area (MPa) (Table 2).

**Table 2:** Key parameters and formulas for shear bond strength calculation

Parameter	Formula/Value
Shear Bond Strength	F/S
F (Load at Failure)	Newton (N)
S (Surface Area)	S = π × r²
	FRC substructure–veneering resin bonding interface (mm^2^)
π (Pi)	3.14
r (Radius)	The diameter of the veneer cylinder (mm)
SBS Unit	MPa

Finally, fracture analysis was conducted using a high-magnification dental microscope (Zumax Medical Co., Suzhou New District, China) at 20× magnification. Failure modes were classified as: adhesive (failure at the interface between core and veneer), cohesive (failure within either the core or veneering material), or mixed (combination of adhesive and cohesive failure).

### Statistical analysis

Normality of data distribution was assessed using the Shapiro–Wilk test, and homogeneity of variances was evaluated with Levene’s test (*p* > 0.05). Shear bond strength (SBS) values were analyzed by two-way ANOVA to test the main effects of veneering material and MLG-FRP framework, as well as their interaction, followed by Tukey’s post-hoc comparisons (α = 0.05). Effect sizes were reported as partial η² for ANOVA main effects and interactions, and as Hedges g (with 95% confidence intervals) for pairwise contrasts (CE vs. CS/VS within each framework).

Two complementary approaches were used: (i) intention-to-treat (ITT), assigning 0 MPa to PTFs, and (ii) per-protocol (PP), excluding PTFs. Kaplan–Meier survival analysis with log-rank tests compared veneer groups, while Cox regression was applied to estimate hazard ratios (CS, VS vs. CE).

All statistical analyses were performed using IBM SPSS Statistics (v26; IBM Corp., Armonk, USA) and Python (Google Colab environment). The Colab script and read-me for the ITT Hedges g analysis are provided in the Supplementary Files (S1–S2) to ensure reproducibi lity.

## Results

A total of 84 specimens were tested, divided equally among the veneering groups. Thirteen premature occurred, exclusively within the hybrid ceramic groups (T-CS: *n* = 4; T-VS: *n* = 4; Z-CS: *n* = 4; Z-VS: *n* = 1). These were assigned an SBS value of 0 MPa in the primary ITT analysis; a PP sensitivity analysis was additionally conducted with PTFs excluded.

It is not uncommon for several specimens to fracture during sample preparation before undergoing stress testing. According to Roulet, PTFs should be handled in two ways: either by assigning them a value of zero or by excluding them from statistical analyses. In the present study, a zero value was assigned to PTFs to ensure that the overall statistical interpretation accounts for their detrimental impact on material performance [18].

### Shear bond strength analysis

Two-way ANOVA indicated a significant main effect of veneering material on SBS (*p* < 0.05), while neither the MLG-FRP framework type nor the interaction reached significance (Table 3).

**Table 3:** Two-way ANOVA results both ITT and PP Anlysis

Source	df	Mean Square	F	*p*-value	partial η²
ITT analysis (0 included)					
MLG-FRP	1	2.96	0.12	0.722	0.002
Veneer	2	132.04	5.66	0.005 *	0.127
MLG-FRP -veneer interaction	2	2.79	0.12	0.887	0.003
PP analysis (0 excluded)					
MLG-FRP	1	5.83	1.06	0.307	0.016
Veneer	2	23.66	4.3	0.018 *	0.117
MLG-FRP -veneer interaction	2	10.66	0.93	0.152	0.056

* indicates significant difference in veneering materials

In both ITT and PP analyses, composite resin (CE) exhibited significantly higher SBS values than both hybrid ceramics (CS, VS). Mean values with 95% confidence intervals for each veneer type and framework are summarized in Table 4.

**Table 4:** The shear bond strength results ITTvs. Per-Protocol analysis

		MLG-FRP Materials			
		Trinia	Zantex		
	Veneer	mean ± SD, 95%CI, *n*	mean ± SD, 95%CI, *n*	Total	*p* values
ITT Analysis (PTFs = 0)	*CE*	12.53 ± 2.89^Aa^ 95%CI:10.86–14.20 *n* = 14	13.21 ± 2.47^Aa^ 95 CI:11.78–14.64 *n* = 14	12.87 ± 2.66^A^ 95 CI:11.88–13.86 *n* = 28	*p* < 0.05*
	*CS*	9.37 ± 6.31^Bb^ 95%CI:5.73–13.02 *n* = 14	9.02 ± 6.24^Bb^ 95%CI:5.41–12.63 *n* = 14	9.19 ± 6.16^B^ 95 CI: 6.91–11.48 *n* = 28	0.991
	*VS*	8.63 ± 5.82^Bb^ 95%CI:5.27–11.99 *n* = 14	9.42 ± 3.54^Bb^ 95%CI:7.38–11.47 *n* = 14	9.02 ± 4.74^B^ 95 CI: 7.27–10.79 *n* = 28	
	*Total*	10.17 ± 5.38 95 CI:8.55–11.8 *n* = 42	10.55 ± 4.68 95 CI:9.14–11.97 *n* = 42	10.36 ± 5.01 95 CI:9.29–11.44 *N* = 84	
PP Analysis (PTFs excluded)	*CE*	12.53 ± 2.89^Cc^ 95%CI:10.86–14.20 *n* = 14	13.21 ± 2.47^Cc^ 95 CI:11.78–14.64 *n* = 14	12.87 ± 2.66^C^ 95%CI:11.88–13.86 *n* = 28	*p* = 0.016*
	*CS*	13.11 ± 1.71^Cc^ 95%CI:11.89–14.35 *n* = 10	12.62 ± 2.39^Cc^ 95%CI:10.91–14.34 *n* = 10	12.87 ± 2.04^C^ 95%CI:11.92–13.83 *n* = 20	
	*VS*	12.08 ± 1.63^Dd^ 95%CI:10.91–13.25 *n* = 10	10.15 ± 2.36^Dd^ 95%CI:8.72–11.58 *n* = 13	10.99 ± 2.25^D^ 95%CI:10.01–11.97 *n* = 23	*p* = 1.00
	*Total*	12.57 ± 2.23 95%CI:11.79–13.35 *n* = 34	11.97 ± 2.72 95%CI:11.07–12.89 *n* = 37	12.26 ± 2.50 95%CI:11.67–12.85 *n* = 71	

Values with different uppercase letters within the same column are significantly different (*p* < 0.05). Values with different lowercase letters within the same row are significantly different (*p* < 0.05)

**Fig. 2:**
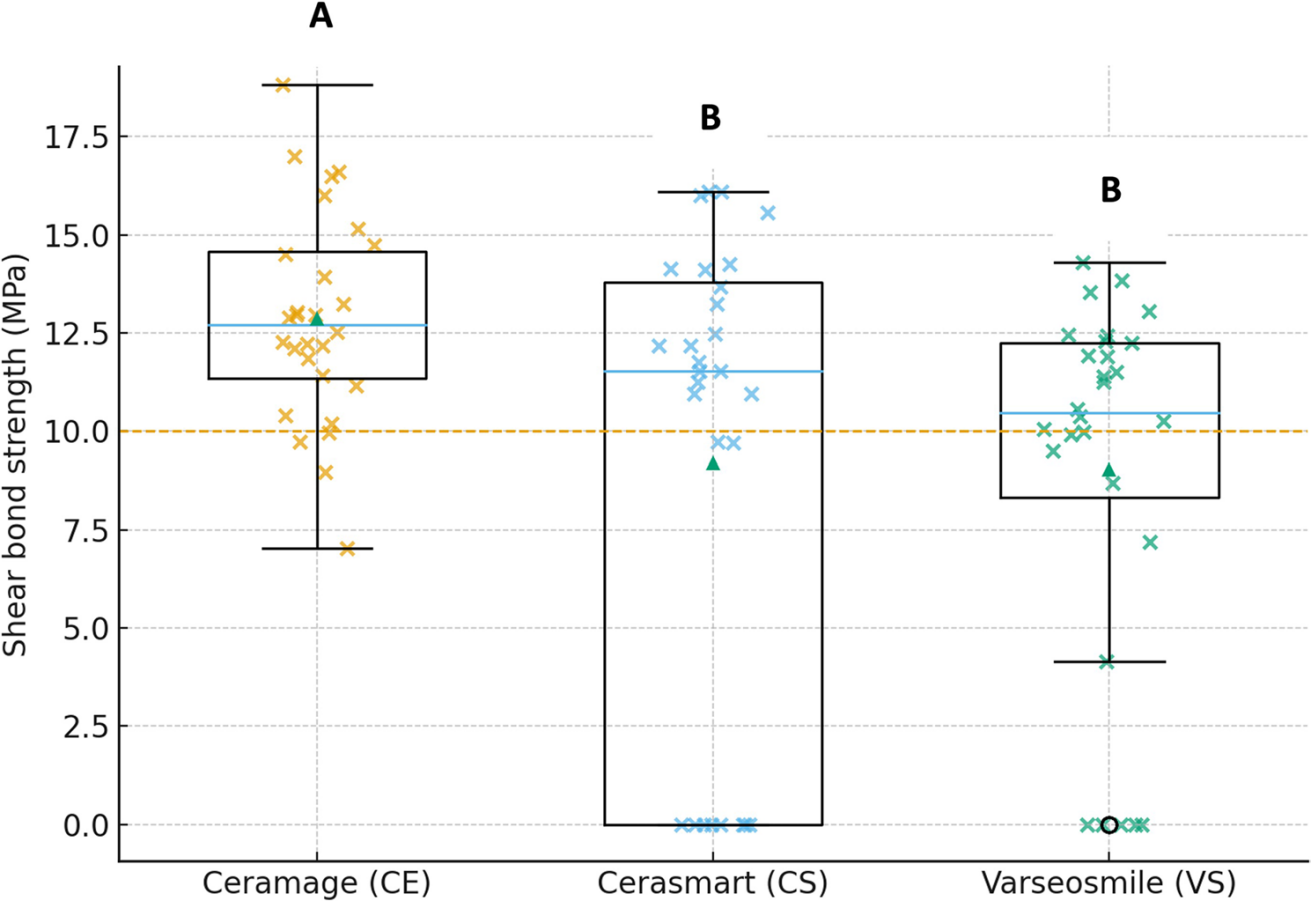
Shear bond strength (MPa) of veneering materials (CE, CS, VS). Dashed horizontal line indicates the 10 MPa reference threshold. Different letters above the boxes denote statistically significant differences between gropus (*p* < 0.05)

Representative boxplots with jittered points and Tukey groupings are shown in Fig. 2, with the 10 MPa clinical threshold line indicated.

Pairwise effect sizes (Hedges g) confirmed moderate-to-large contrasts between CE and hybrid ceramics across both MLG-FRP frameworks (Table 5).

**Table 5:** Pairwise effect (Hedges g) sizes for shear bond strength comparisons of CE vs CS and CE vs VS within each MLG-FRP. Superscript letters denote Tukey's HSD grouping from the two-way ANOVA (Table 4)

MLG-FRPs	Comparison	*n*	Mean ± SD (CE)	Mean ± SD (Others)	Hedges-g	95% CI
Trinia	CE vs. CS	14	12.53 ± 2.89	9.37 ± 6.31^Bb^	0.62	−0.14-1.38
Trinia	CE vs. VS	14	12.53 ± 2.89	8.63 ± 5.82^Bb^	0.82	0.05–1.60
Zantex	CE vs. CS	14	13.21 ± 2.47	9.02 ± 6.24^Bb^	0.86	0.08–1.63
Zantex	CE vs. VS	14	13.21 ± 2.47	9.42 ± 3.54^Bb^	1.2	0.39–2.01

### Survival analysis

**Fig. 3:**
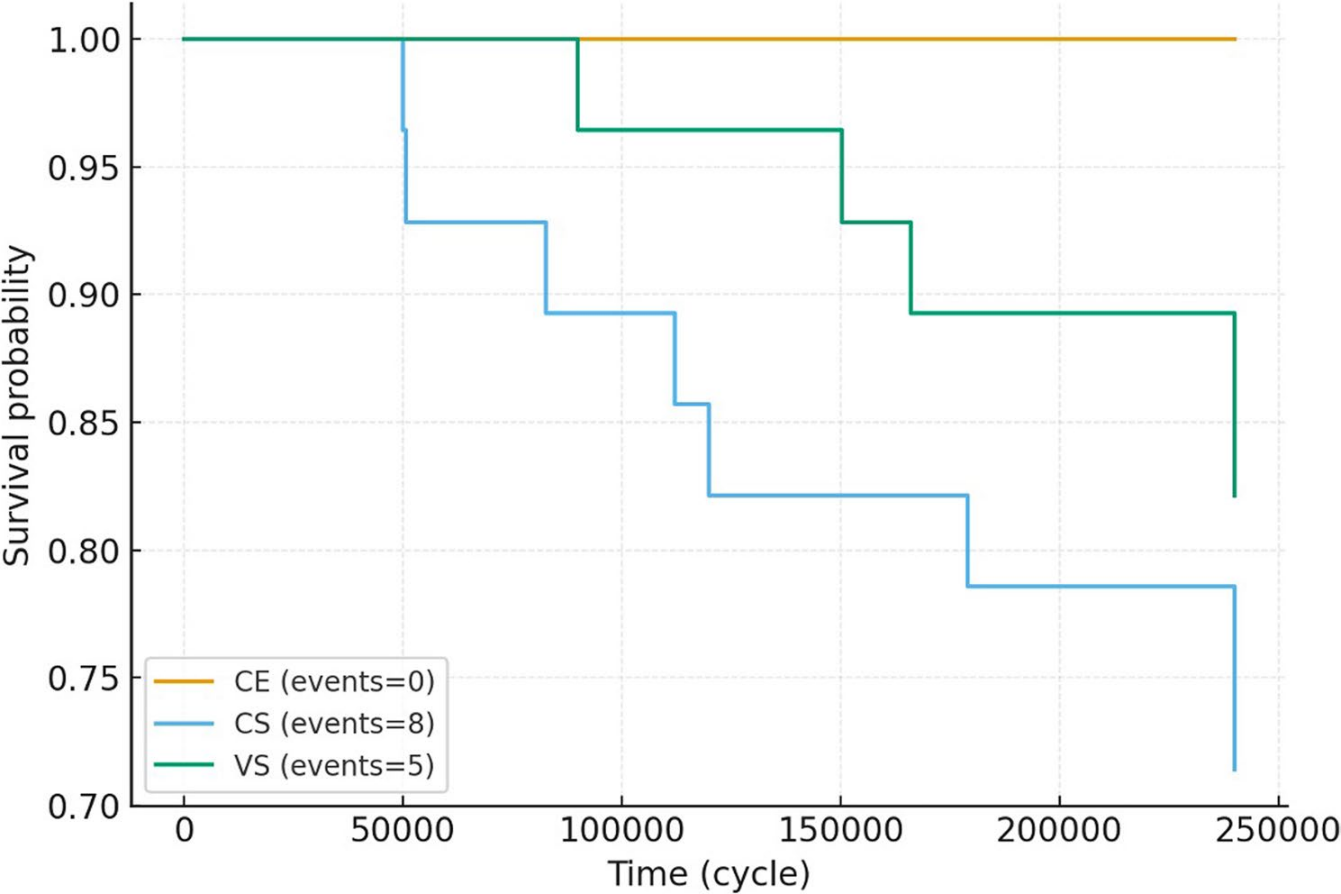
Survival curves (Kaplan–Meier) of veneering groups after thermo-mechanical aging. Composite resin (CE) maintained superior survival compared with hybrid ceramic groups (CS, VS)

Kaplan–Meier survival analysis demonstrated distinct differences among veneer groups (Fig. 3).

The CE group showed 100% survival throughout aging, while CS and VS groups exhibited stepwise reductions. The log-rank test revealed statistically significant overall differences (*p* < 0.05, Table 6).

**Table 6:** Kaplan–Meier survival analysis results for veneering groups

Veneer	*n*	Events ^a^	Median Survival Time (Cycle)	Survival at 240,000 cycles	Log-rank *p*-values (pairwise)
*CE*	28	0	240,000(NE*)	100%	CE vs. CS:0.002 CEvs VS: 0.02
*CS*	28	8	240,000	< 100%	CSvs VS: 0.36
*VS*	28	5	240,000	< 100%	

^a^ first occurrence of debonding or fracture during scheduled failure checks

*NE: Non-estimable, as no failures occurred in CE group

Pairwise comparisons showed CE significantly outperformed both CS and VS, whereas no difference was observed between CS and VS. In Cox regression, CE yielded no events; therefore, hazard ratios relative to this group were non-estimable.

### Failure mode analysis

**Fig. 4:**
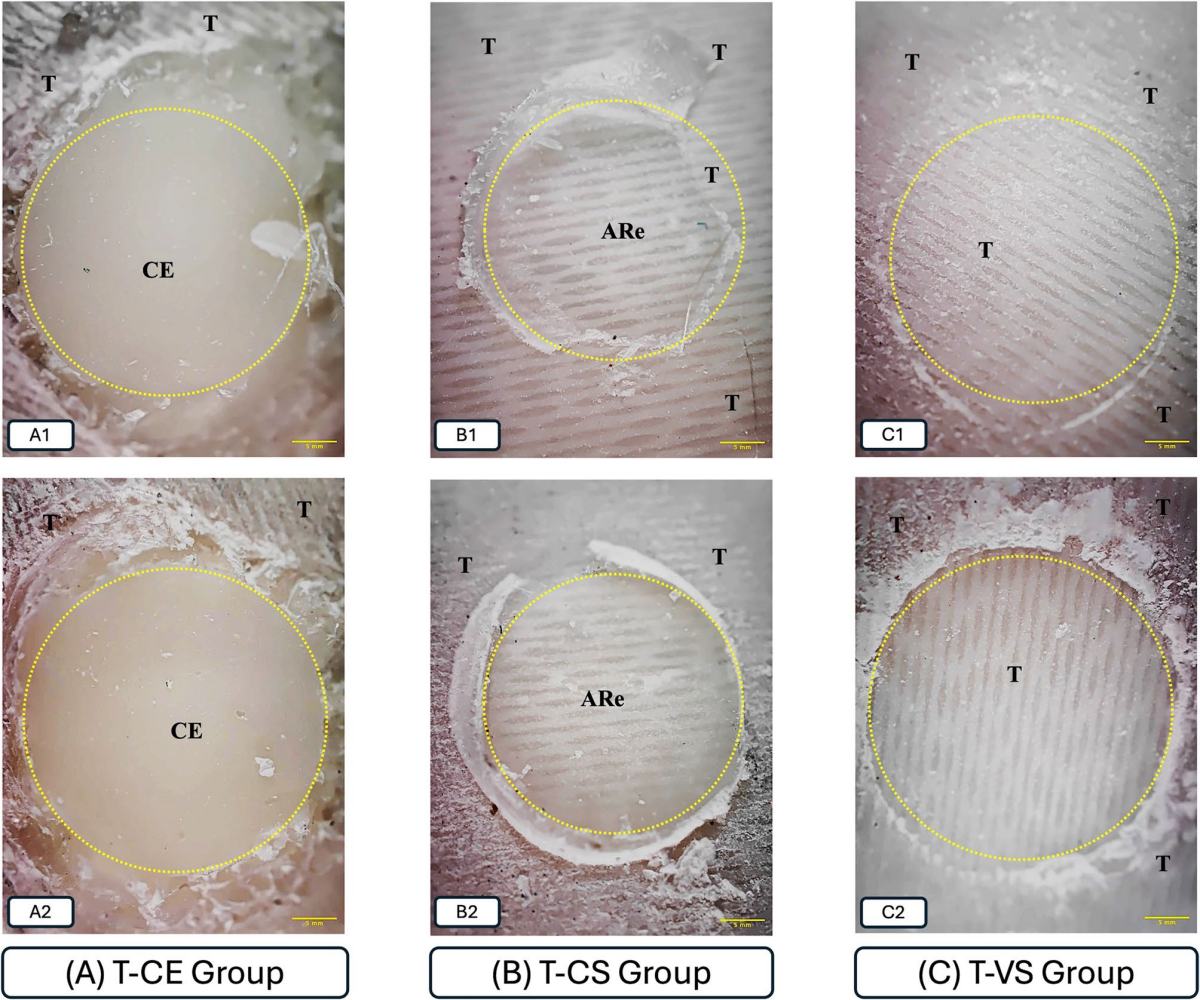
Representative images of the failure modes of the shear test in Trini groups (20x). T: trinia, CE: ceramage, CS: cerasmart, VS: VarseoSmile Crown Plus, ARe: adhesive resin. The dotted loops indicate regions corresponding to a 7 mm^2^ bonding area. **A**: T-CE group, (A1-A2) Cohesive failure occurred within the composite resin (CE) itself. **B**: T-CS group, (B1) Adhesive failures were observed at the bonding interface, where the adhesive resin separated from the substrate—occurring in some areas between the resin and Trinia, and in others between the resin and CeraSmart. (B2) Adhesive failures were observed at the bonding interface between the resin and CeraSmart. **C**: T-VS group, (C1-C2) Adhesive failure occurred at the interface between Trinia and adhesive resin

**Fig. 5:**
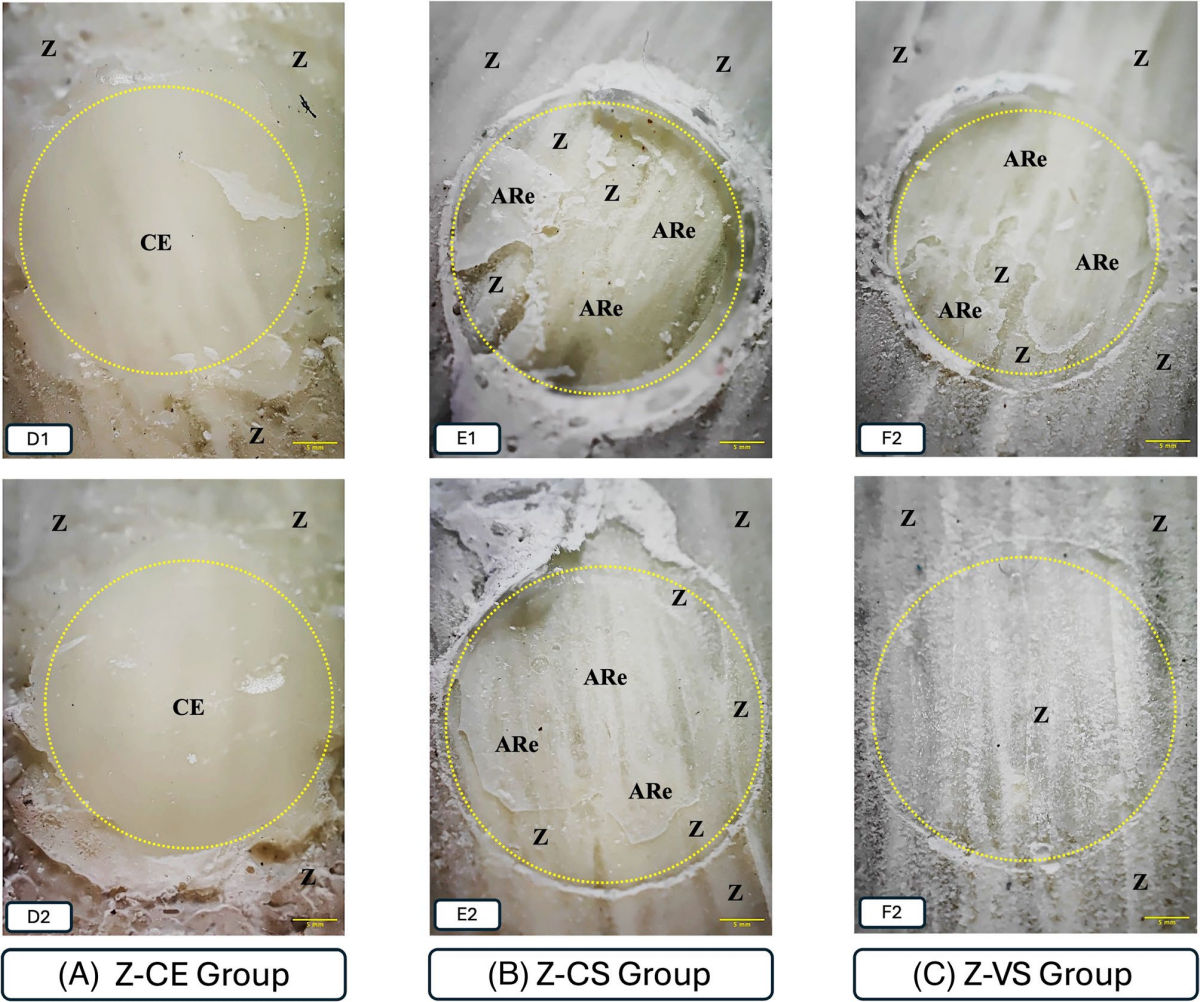
Representative images of the failure modes of the shear test in Zantex group (20x). Z: zantex, CE: ceramage, CS: cerasmart, VS: VarseoSmile Crown Plus, ARe: adhesive resin. The dotted loops indicate regions corresponding to a 7 mm^2^ bonding area. **A**: Z-CE group, (D1-D2) Cohesive failure occurred within the composite resin itself. **B**: Z-CS group, (E1-E2) Adhesive failures were observed at the bonding interface, where the adhesive resin detached from the substrate—occurring in some regions between the resin and Zantex, and in others between the resin and CeraSmart. **C**: Z-VS group, (F1) Adhesive failures were observed at the bonding interface, where the adhesive resin detached from the substrate—occurring in some regions between the resin and Zantex, and in others between the resin and VarseoSmile Crown Plus. (F2) Adhesive failure occurred at the interface between the adhesive resin and Zantex

Fractographic analysis revealed that failures were predominantly adhesive across all groups (> 90%). Cohesive failures occurred only in CE specimens, within the veneering composite itself. Failure mode distributions are illustrated in Figs. 4 and 5.

**Fig. 6:**
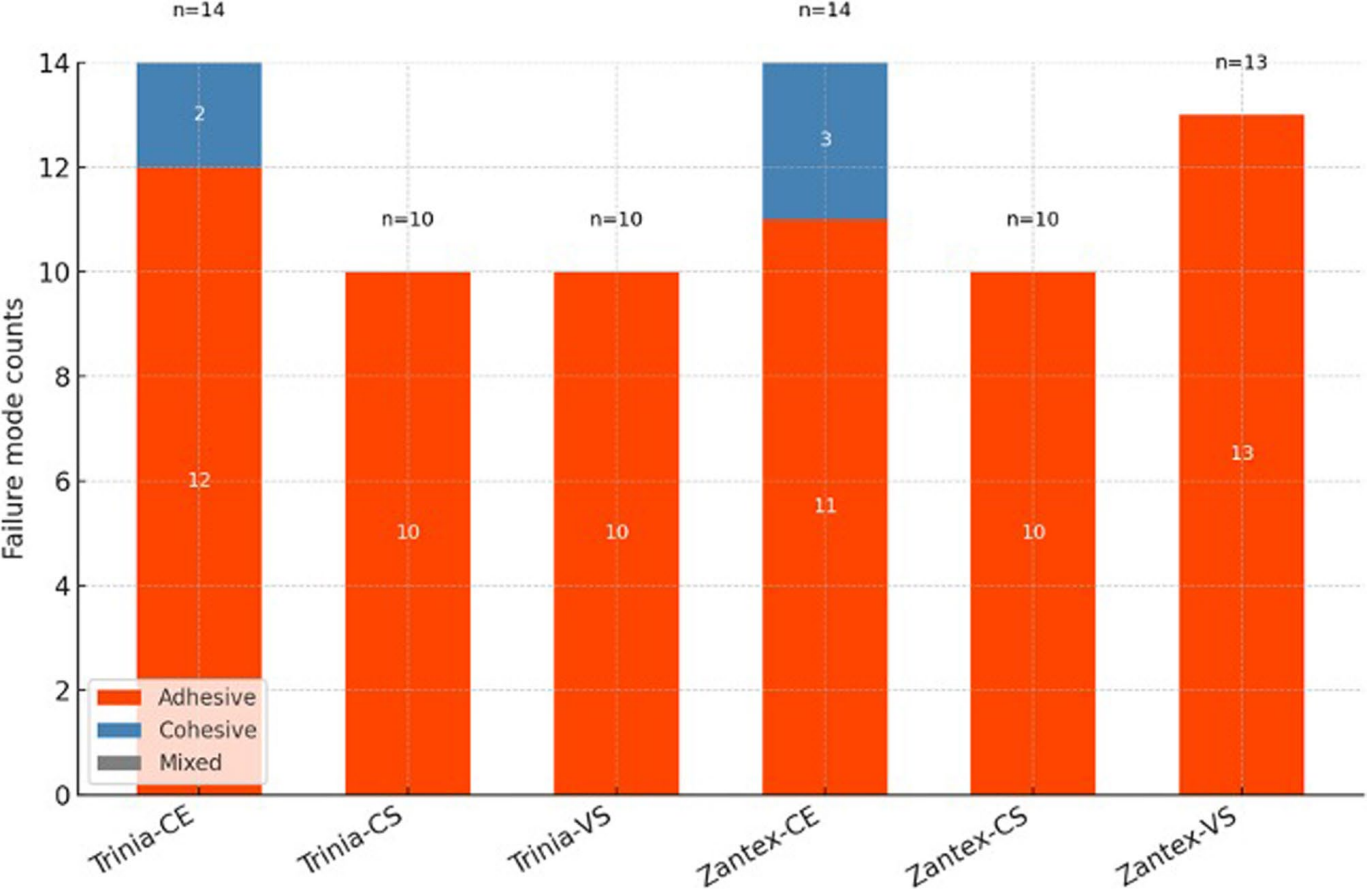
Failure mode distribution across groups

Adhesive failures were predominant across all groups. However, a limited number of cohesive failures were observed in the composite resin veneering group (Ceramage), specifically three in the Zantex group (21.42%) and two in the Trinia group (14.28%). No mixed failure patterns were detected (Fig. 6).

## Discussion

The findings of the present study refute the initial hypothesis that milled, or 3D-printed hybrid ceramic materials would demonstrate superior bond strength to MLG-FRP frameworks compared to composite resins. The results clearly indicate that the choice of veneering material affects the bonding performance of restorations built on MLG-FRP substructures.

Dental restorations face various intraoral stresses, including salivary exposure, thermal fluctuations, chemical agents, and masticatory forces. These factors contribute to material degradation and failure over time. While clinical studies provide valuable insights into long-term adhesive success, they often cannot isolate specific factors influencing bond durability [19]. Therefore, standardized laboratory tests are essential for rapid and reproducible evaluation of these variables under controlled conditions [20]. When comparing different studies, it is crucial to consider key aspects such as aging protocols, bond strength measurement methods, and material properties.

New biomaterials are routinely subjected to aging and fatigue methods to assess their compatibility with the complexities of human oral physiology [21]. Thermocycling and mechanical fatigue tests are commonly applied in studies to simulate clinical service [22], and these procedures have a marked impact on adhesive performance [23]. In the present study, specimens underwent 5000 thermocycles between 5 and 55 °C and 240,000 mechanical cycles, approximating one year of mastication with an average bite force of 50 N [17]. Then, specimens were tested in a water medium [24].

Bond strength is a crucial factor influencing the structural integrity of MLG-FRP-based prostheses. It determines the durability of the framework-veneer interface, which is often the system’s susceptible point. Despite its value, bond strength testing lacks universal standardization and carries some limitations [25]. Macro-tests like shear and tensile often result in cohesive failures and may overestimate values, leading to the development of micro-tests for more accurate results. Although micro-tests improve reliability, they remain technique-sensitive [26]. Various factors, including specimen preparation, substrate properties, and testing methodology, influence bond strength results [27]. Consistent with previous research, this study used a macro shear test due to its ease and ability to produce clear results with uniform stress distribution [13]. This methodological decision reflects clinically relevant conditions in which extensive bonded interfaces with a 7 mm^2^ bonding area were exposed to repetitive masticatory loads.

This study compared the bonding performance of Zantex and Trinia, examining conventional composite resin alongside milled and 3D-printed ceramic-containing hybrid materials. These alternatives are known for their excellent physical properties in full-coverage crowns [28] and were tested to address failure scenarios often seen with conventional composites in MLG-FRPs. The results indicate that composite resin offers superior SBS compared to milled hybrid nanoceramic and 3D-printed hybrid composite resin, regardless of the MLG-FRP substructure materials [29]. Under the present aging conditions, only the CE achieved bond strengths above the 10 MPa clinical threshold, while CS and VS did not, suggesting that composite resin may offer superior long-term reliability. As Kitasako et al. [30] noted, shear bond strength is material-dependent and correlates with elastic properties. The superior performance of composite resin veneers may be partly explained by the elastic modulus compatibility between the resin matrix and the FRP core, which reduces interfacial stress and promotes more uniform load distribution [31]. In contrast, the stiffer hybrid ceramics may generate higher stress concentrations at the veneer–framework interface, predisposing to debonding under fatigue.

In contrast, the comparatively rigid structure of ceramic-containing hybrid materials may generate higher interfacial stress, promoting micro-gap formation and bond strength loss. It should be noted that different bonding strategies were used: Ceraresin Bond I/II for CE and Panavia V5 for CS/VS. This methodological variability may have favored CE, and the superior performance observed should therefore be interpreted with caution. Future studies should employ a single universal bonding protocol to better isolate the effect of veneer type.

To the authors’ knowledge, this study is the first to investigate bonding between milled (GC CeraSmart) and 3D-printed (VarseoSmile Crown Plus) hybrid ceramics and MLG-FRPs. Thus, direct comparison with prior studies is limited. However, findings from Graf et al. [32] offer partial context. They evaluated retention of 3D-printed (VarseoSmile Crown Plus) and milled (Enamic and Lava Ultimate) crowns bonded to Trinia abutments after aging. They found 3D-printed crowns had lower retention than milled ones. In this study, VarseoSmile Crown Plus also showed the lowest bond strength, aligning with Graf et al.’s findings, but no statistically significant difference was detected between milled and 3D-printed hybrids for either core material. Graf et al. reported adequate pull-off values for clinical use, while here both groups showed bond strengths slightly below the acceptable limit. The composite resin SBS aligned well with previous FRP studies [7, 12, 33]. Although hybrid ceramics showed lower SBS values in this study, this outcome should not be interpreted as an inherent material weakness. Their bonding performance is strongly protocol-dependent and may be influenced by surface treatment, adhesive selection, filler composition/silanation, and modulus mismatch with the polymer framework. Different bonding strategies might yield improved results.

Moreover, early catastrophic failures (PTFs) observed in the hybrid ceramic groups should be interpreted clinically as “no usable bond.” In line with this, both ITT analysis and per-protocol sensitivity analysis were reported to provide complementary perspectives. Kaplan–Meier survival analysis further illustrated the temporal pattern of failures, demonstrating superior survival of CE compared to CS and VS. These findings underline the need for protocol optimization to reduce the risk of early debonding events, which are particularly critical for clinical longevity.

Failure mode analysis revealed that adhesive failures dominated across all groups (approximately 94.1% adhesive and 5.95% cohesive failures), highlighting potential limitations in the current surface conditioning and bonding protocols, particularly for hybrid ceramic groups. This predominance of adhesive failures, especially among the hybrid ceramic groups, suggests that current surface conditioning and bonding strategies may be inadequate for ensuring durable adhesion between hybrid ceramics and MLG-FRP frameworks. The use of universal adhesives and MDP-containing cements, while effective in some contexts, may not provide sufficient chemical interaction with the hybrid ceramic surfaces after aging, particularly given their lower glass filler content and polymeric matrix, which can limit silane or functional monomer interaction. These findings underscore the necessity of optimizing bonding protocols specifically tailored for hybrid ceramic materials, possibly through alternative surface pre-treatments or novel adhesive formulations.

This observation is consistent with AlJehani et al. [34], contrasting with studies that reported a higher incidence of cohesive failures within the substrate [22]. Matinlinna et al. demonstrated that silane improves fiber-resin adhesion, which was confirmed by our protocol for conventional composite resin [35].

The silane agent used in this study enhances the bond between the fibers and the resin matrix, theoretically leading to strong adhesion. However, the frequent adhesive failures observed across all FRC groups suggest that the bonding interface may be largely influenced by the polymer surface areas without glass fibers. MLG-FRP surfaces consist of smooth polymer areas without exposed fibers and roughened areas with exposed glass fibers. The heterogeneity of fiber-reinforced CAD/CAM polymers such as Trinia and Zantex arising from variations in glass fiber content, orientation, and polymer matrix volumemay significantly influence their bonding behavior. In our study, the bonding surfaces were prepared through standardized surface treatment protocols; however, due to the intrinsic structure of these materials, regions with fiber-rich and those dominated by matrix-rich may have been unevenly distributed. This topographical inconsistency may have contributed to the observed adhesive failures, particularly at the interface. The observed decrease in bond strength after fatigue cycling further indicates possible long-term limitations that warrant attention. Additionally, the occurrence of 13 premature failures in the hybrid ceramic groups is a notable finding prior to the fatigue protocol. The inability to form an initial bond in the hybrid ceramics suggests that, without optimized bonding strategies, these materials may present a higher risk of early clinical failure.

The analysis indicated that the luting procedure might have played a key role in determining the bond between the framework and the veneering material for non-monolithic restorations in this study. Surface pretreatment remains crucial. Airborne-particle abrasion and silanization improve adhesion [13, 36]. In this study, both Trinia and Zantex were air abraded with Al₂O₃ before veneering. Composite resin was bonded to sandblasted surfaces with silane, while hybrid ceramics were luted with Panavia V5 containing 10-methacryloxydecyl dihydrogen phosphate (MDP). Interestingly, MLG-FRPs bonded with MDP showed lower bond strength than those treated with silane [12]. This finding contrasts with reported improvements in glass fiber post-bonding with MDP-containing compounds, suggesting material-specific interactions. The underlying mechanisms remain unclear, particularly how MDP interacts with varied fiber types, volume fraction, orientation and resin matrices (of unspecified composition) in FRPs needs further study [37]. Moreover, MDP-based adhesive systems, such as those used for CS and VS, are susceptible to hydrolytic degradation during water storage and aging, further compromising long-term bond durability [38].

It is important to acknowledge that, like all in vitro studies, this investigation has certain limitations. First, the sample size calculation in our work is based on a surface-treatment study that utilized the same ISO 29022 shear method. This is a proxy because both target the bonded interface. Observed effects (F = 5.66, *p* = 0.005, partial η²=0.127; Hedges’ g ≈ 0.62–1.20) indicate the sample was adequate. Second, a non-aged control group was deliberately omitted, as intraoral conditions inevitably subject dental prostheses to aging. The primary aim was to assess the bonding performance of multilayer fiber-reinforced polymer (FRP) substructures with various veneering materials under simulated aging, enhancing the clinical relevance of the findings. Another limitation is the use of different adhesive protocols for the tested veneering materials (silane-based bonding for CE vs. MDP-based luting for CS and VS. This methodological choice may have contributed to the higher SBS values in the CE groups. Future investigations should apply a standardized bonding strategy across all veneer types to exclude adhesive variability as a confounding factor. Additionally, long-term in vivo or in situ studies are needed to validate these results in clinical settings.

Within the limitations of this in vitro study, composite resin veneers appear to be the most reliable choice for veneering FRP frameworks in load-bearing regions, as they consistently surpassed the 10 MPa clinical threshold. Hybrid ceramic veneers, although offering superior esthetics and polishability, demonstrated lower bond strength under the tested protocols and should be used cautiously in areas subjected to lower functional stress. Their broader clinical application may become feasible once optimized bonding strategies are developed and validated.

## Conclusion

Within the limitations of this in vitro study, the following conclusions can be drawn regarding material selection and bond strength:

1. Composite resin exhibited higher bonding performance to MLG-FRP frameworks compared to both milled and 3D-printed hybrid ceramics.
2. Only the composite resin veneer exceeded the 10 MPa clinical threshold, whereas both milled and 3D-printed hybrid ceramics showed similar values below this limit.
3. The results indicate that the limited bonding performance of hybrid ceramics is primarily attributable to the adhesive interface.
4. Differences in luting cement systems can notably impact the shear bond strength of MLG-FRP restorations across varying core materials. Therefore, improving the interfacial adhesion strategies is crucial to enhance the clinical reliability of hybrid ceramic veneering on MLG-FRP frameworks.

## Supplementary Information

Supplementary Material 1.

Supplementary Material 2.

Supplementary Material 3.

## Data Availability

All raw data and analysis code are openly available in Zenodo at [**10.5281/zenodo.17273820**] (10.5281/zenodo.17273820) **under a CC BY 4.0 license.**

## Abbreviations

CAD/CAM: Computer-aided design/computer-aided manufacturing
FRP: Fiber-reinforced polymer
MLG-FRP: Multilayer glass-fiber-reinforced polymer
CS: Cerasmart
VS: VarseoSmile Crown Plus
3D: Three dimensional
STL: Standard tessellation language
DLP: Digital light processing
CE: Ceramage
LED: Light-emitting diode
SBS: Shear bond strength
ITT: Intention-to-treat
PP: Per-protocol
HSD: Tukey’s honestly significant difference
PTF: Premature test failures
T: Trinia
Z: Zantex
